# Knowledge, Attitudes, and Practices Toward Weight Management Among Patients with Type 2 Diabetes Mellitus in Saudi Arabia: A Cross-Sectional Study

**DOI:** 10.3390/healthcare13212770

**Published:** 2025-10-31

**Authors:** Miyad Khazna, Abdullah Zaki Al-Fahd, Jomanah Atyah Alatawi, Badriah Almotiri, Remas Abdulrahman Alduwaish, Rahaf Alanazi, Khalid Albedaiwi, Sharfuddin Chowdhury

**Affiliations:** 1 College of Medicine, Alfaisal University, Riyadh 11533, Saudi Arabia; 2 College of Medicine, Imam Muhammad ibn Saud Islamic University, Riyadh 11432, Saudi Arabia; 3 College of Medicine, University of Tabuk, Tabuk 47311, Saudi Arabia; 431006950@stu.ut.edu.sa; 4 College of Medicine, King Abdulaziz University, Jeddah 21589, Saudi Arabia; bbaraktallah@stu.kau.edu.sa; 5 College of Respiratory Care, Almaarefa University, Riyadh 11597, Saudi Arabia; 221220252@student.um.edu.sa; 6 College of Medicine, University of Hail, Hail 55481, Saudi Arabia; s201905767@uoh.edu.sa; 7 Department of Emergency Medicine, King Abdulaziz Specialist Hospital, Al-Jawf 74631, Saudi Arabia; kalbedaiwi@moh.gov.sa; 8 School of Health Professions, University of Alabama at Birmingham, Birmingham, AL 35233, USA; schowdh6@uab.edu; 9 Trauma Center, King Saud Medical City, Riyadh 12746, Saudi Arabia

**Keywords:** knowledge, attitude, practice, diabetes mellitus, weight management, Saudi Arabia, cross-sectional study

## Abstract

**Background/Objectives**: Weight management is crucial for preventing and managing diabetes. This study aimed to evaluate the knowledge, attitude, and practice (KAP) of people with Type 2 Diabetes Mellitus (T2DM) regarding weight management in Saudi Arabia. **Methods**: This cross-sectional survey of patients with T2DM in Saudi Arabia was conducted from October 2024 to February 2025. Participants completed a self-administered online questionnaire, which was validated and adapted from a previous study. **Results**: Among 385 individuals, 60.0% were females, and 34.0% were between 18 and 25 years old. The average scores for knowledge, attitude, and practice were 8.7 ± 3.6 (possible range: 0–13), 28.5 ± 4.4 (possible range: 10–50), and 28.3 ± 4.0 (possible range: 9–45), respectively. Pearson’s correlation analysis shows no significant correlation between knowledge and attitude (*r* = −0.016, *p* = 0.750). However, a weak yet statistically significant positive correlation was found between knowledge and practice (*r* = 0.155, *p* = 0.002). Additionally, a moderate negative correlation was observed between attitude and practice (*r* = −0.353, *p* = 0.001). **Conclusions**: The participants exhibited moderate knowledge, negative attitudes, and moderate practices regarding weight management. These findings provide crucial guidance for developing healthcare strategies to improve the KAP of diabetic patients in weight management.

## 1. Introduction

Diabetes mellitus (DM) is a chronic endocrine disease with multiple causes. It is marked by uncontrolled increases in blood sugar (hyperglycemia) due to impaired insulin secretion, defective insulin function, or both [1]. DM is a growing global health issue with high prevalence. According to the International Diabetes Federation, the number of adults (20–79 years old) with diabetes was 588.7 million in 2024 worldwide. This number is expected to rise to 852.5 million in 2050. Saudi Arabia ranks among the world’s top ten for adult diabetes prevalence. There are more than 5.3 million adults with diabetes, which could increase to as many as 9.5 million by 2050 [2]. DM can lead to several complications, including retinopathy, nephropathy, neuropathy, heart diseases, and increased vulnerability to infections [3].

The risk of developing Type 2 Diabetes Mellitus (T2DM) increases with factors such as excess body weight/obesity, decreased physical activity, a diet high in sugar and low in fiber, smoking, and a family history of DM [4]. Prevention and management are extremely important. Weight reduction plays a key role in preventing diabetes, helping achieve clinical remission, and reducing the risk of complications. A sustained weight loss of at least 15% can significantly slow the progression of T2DM, lead to remission in many patients, and greatly improve metabolic health in others [5]. Intensive behavioral and lifestyle programs, weight loss medications, and bariatric surgeries can result in long-term, meaningful weight loss and lower obesity-related risks. Even modest weight loss can improve glycemic control and reduce the need for glucose-lowering medications, while greater weight loss can lead to significant reductions in Hemoglobin A1c (HbA1c) and fasting blood glucose (FBG) [6]. In addition, intensive lipid-lowering in the T2DM population, alongside optimized glycemic control, is associated with regression of atherosclerotic plaque, highlighting the importance of structured weight management in cardiovascular risk reduction [7].

Awareness of weight regulation and its role in controlling diabetes is essential for lowering the risk of the disease. Prior research from China in Qidong City showed that diabetic patients had moderate knowledge, neutral attitudes, and inappropriate practices toward weight management [8]. Additionally, studies in Iran and South Africa suggested that diabetic patients have a relatively low awareness of the benefits of weight management [9,10]. Although Saudi Arabia ranks among the countries with the highest adult DM prevalence, there are no KAP studies on weight management among the diabetic population. Therefore, this study aims to assess the KAP of diabetic patients regarding weight management in Saudi Arabia.

## 2. Materials and Methods

### 2.1. Study Design

We conducted a cross-sectional study by enrolling diabetic patients in Saudi Arabia through convenience sampling between October 2024 and February 2025.

### 2.2. Study Subjects

The inclusion criteria for this study are (1) patients diagnosed with T2DM; (2) individuals aged 18 years or older; and (3) those who agreed to participate in the study. The exclusion criteria are (1) patients with no or limited legal capacity; (2) individuals diagnosed with untreated mental illnesses; (3) individuals with reading or comprehension difficulties; (4) those affected by cancer or significant organ dysfunction that prevents independent self-care and daily activities; and (5) pregnant women.

### 2.3. Study Sample

A minimum of 385 participants were required based on the Raosoft sample size calculator (95% confidence level, 5% margin of error). The final analytic sample included 385 participants.

### 2.4. Sampling Technique, Data Collection Method, and Instrument Used

This study used a self-administered online questionnaire for all participants who met the criteria. The questionnaire was validated and adapted from a previous study [8]. We translated it into Arabic, the local language in Saudi Arabia. A data collector was assigned to help reach the target number of participants. The questionnaire consisted of 45 items, divided into four sections: demographic characteristics, knowledge, attitude, and practice toward weight management.

Twelve demographic characteristics were included: age, gender, educational level, medical-related occupation, physical labor occupation, monthly income, medical insurance, course of T2DM, diabetic medications, hyperlipidemia, fatty liver, and screening for excess visceral fat.

The other sections of the questionnaire were as follows: The knowledge section included 14 questions. Correct answers to questions 1–12 and 14 earned 1 point each, while incorrect or unclear answers earned 0 points. Additionally, the questionnaire’s validity was evaluated using question 13. The total score could range from 0 to 13. The attitude section consisted of 10 questions rated on a five-point Likert scale. Items 2–4 were positively worded and scored from “strongly agree” (5 points) to “strongly disagree” (1 point), while the other items were negatively worded. The possible score range was from 10 to 50. The practice section had nine questions, also rated on a five-point Likert scale. Questions 1, 2, 5, 6, 7, and 9 were positively phrased; the remaining questions were negatively phrased. The total score range was from 9 to 45.

Based on their scores in each section, participants were divided into three levels: (80–100%), indicating strong knowledge, positive attitudes, and proactive practices; (60–79%), reflecting moderate knowledge, neutral attitudes, and moderate practices; and (<60%), signifying inadequate knowledge, negative attitudes, and inappropriate practices. Cronbach’s alpha was used to assess internal consistency for knowledge, attitude, and practice.

### 2.5. Ethical Consideration

The study received approval from the King Saud Medical City Institutional Review Board (IRB), reference number H1RI-12-Sep24-02. We explained the study’s aims to the participants before obtaining their consent. Data collection and access will be limited to the principal investigator, biostatistician, and data collectors.

### 2.6. Statistical Analysis

Data analysis was performed by an experienced biostatistician using Statistical Package for the Social Sciences (SPSS) version 27 (IBM Corp., Armonk, NY, USA, 2020). Descriptive statistics, including means and standard deviations, were used to summarize participants’ demographic and health-related characteristics.

To examine the differences in KAP scores across various factors, one-way analysis of variance (ANOVA) was used for factors with more than two categories, and independent t-tests were employed for factors with two categories. Pearson’s correlation analysis was performed to assess the relationships between KAP dimensions. A *p*-value of less than 0.05 was considered statistically significant for all analyses.

## 3. Results

### 3.1. Demographic Characteristics

Among 385 participants, the largest age group was 18–25 years old (131, 34.0%). More females participated in the study (231, 60.0%). Regarding education level, most had completed junior college or higher (261, 67.8%). Regarding employment, most patients were not employed in a medical-related occupation (321, 83.4%) or engaged in physical labor (231, 60.0%). Concerning monthly income, the highest percentage earned less than 5000 Saudi Riyals (173, 44.9%). Regarding medical insurance, most patients were uninsured (164, 42.6%). The majority had been diagnosed with T2DM for less than a year (230, 59.7%). When it came to medication for blood glucose control, most patients were taking oral hypoglycemic drugs (160, 41.6%). Additionally, many patients had not been diagnosed with hyperlipidemia (335, 87.0%) or fatty liver (322, 83.6%). Lastly, most had not undergone screening for excess visceral fat (305, 79.2%).

### 3.2. Knowledge

The most frequently correct answers included the following items: reducing body weight and fat content improves insulin resistance symptoms (294, 76.4%), restricting calories and exercising regularly increases energy expenditure (310, 80.5%), and weight loss helps lower blood sugar, blood pressure, and lipid levels (308, 80.0%). Conversely, the least correct response involved the statement “BMI falls within 18.5~24 kg/m^2^, while those above 24 are classified as overweight, and those above 28 are considered obese,” with only 150 (39.0%) patients answering correctly. The total score for participants in this study, which ranged from 0 to 13, had an average of 8.7 ± 3.6. Cronbach’s alpha for knowledge was 0.824.

### 3.3. Attitudes

The most frequently endorsed item was “Achieving weight management requires greater effort on my part compared to healthy individuals” (184; 47.8%), followed by “Diabetes significantly increases my susceptibility to obesity compared to the general population” (158; 41.0%). The lowest endorsement was for “Weight loss adversely affects my resistance levels,” and “I perceive no need for weight loss if I only have a slight excess of visceral fat and a normal BMI,” with 87 participants (22.6%) strongly agreeing with each statement. Total attitude scores ranged from 19 to 43, with a mean score of 28.5 ± 4.4. Cronbach’s alpha for attitudes was 0.856.

### 3.4. Practices

The overall score ranged from 15 to 38, with a mean score of 28.3 ± 4.0. The most commonly reported practice was “Carbohydrate intake in my daily diet will be monitored,” with 33.0% of patients stating they “Always” monitor their carbohydrate intake, followed by 31.2% who “Frequently” monitor it. For the sentence “daily weight management involves engaging in resistance exercises and aerobic,” 31.2% of patients reported doing so “Always,” and 33.5% reported doing so “Frequently.” On the other hand, the least common practice was “I use medication (e.g., orlistat, metformin) for weight management,” with 18.7% of patients reporting “Never” use. However, 28.1% reported using it “Sometimes,” and 25.2% reported using it “Frequently.” Regarding surgery, the statement “If lifestyle changes and medications prove ineffective in weight management, I may consider surgery” had 24.7% of patients saying they would “Always” consider surgery, with 26.2% saying they would do so “Frequently.” Cronbach’s alpha for practices was 0.889.

### 3.5. Factors Affecting Patients’ KAP

Referring to (Table 1), in the Knowledge Domain, the group aged 35–55 had the highest knowledge scores (9.55 ± 3.43, *p* = 0.001), indicating better knowledge about weight management compared to younger and older patients. Additionally, those using oral hypoglycemic drugs had the highest knowledge scores (9.94 ± 2.94, *p* = 0.001), while those not on medication had the lowest scores. Other statistically significant factors include monthly income, the duration of the DM course, hyperlipidemia, and screening for excess visceral fat. In the Attitude Domain, uninsured individuals had the highest attitude scores (29.37 ± 4.65, *p* = 0.002), whereas patients diagnosed with fatty liver had lower scores (27.52 ± 3.98, *p* = 0.049). Gender, occupation in physical labor, and screening for excess visceral fat were also statistically significant. In the Practice domain, most demographic and clinical factors showed no significant association with practice scores, indicating broadly similar weight management practices across groups. Only a few variables showed modest differences: educational level (*p* = 0.029), medication type overall (*p* = 0.049), and fatty liver diagnosis (*p* = 0.042).

### 3.6. Pearson Correlation Analysis of KAP Dimensions

According to Table 2, the Pearson correlation analysis shows no significant relationship between knowledge and attitude (*r* = −0.016, *p* = 0.750). However, a weak but statistically significant positive relationship was found between knowledge and practice (*r* = 0.155, *p* = 0.002). Conversely, a moderate negative relationship was observed between attitude and practice (*r* = −0.353, *p* = 0.001).

## 4. Discussion

The findings of this study showed that diabetic patients in Saudi Arabia had moderate knowledge, negative attitudes, and moderate practices regarding weight management based on data from 385 participants. Consistent with our results, previous research from China indicates that diabetic patients had moderate knowledge, neutral attitudes, and inappropriate practices toward weight management [8]. Additionally, studies in Bangladesh revealed that many individuals with T2DM lacked awareness of healthy body weight, caloric needs, and proper methods of weight measurement [11]. Also, a study of diabetic patients in Ethiopia found only moderate knowledge and neutral attitudes, with limited adoption of lifestyle changes such as healthy eating, moderate weight loss, and exercise [12]. Several factors can lead to cross-country variation in KAP. For example, cultural differences like Ramadan fasting in the Muslim population can influence self-management behaviors and glycemic control in diabetics [13]. Health care access could also play a role. In Saudi Arabia, the government provides free diabetic care for its citizens in the public hospitals and medical centers [14]. On the other hand, diabetic people in Bangladesh have difficulties accessing healthcare centers, where only 33.5% were diagnosed, and only 24.6% received treatment [15]. Overall, these findings emphasize the need to improve weight management education for diabetic patients to address KAP gaps.

The average knowledge score was (8.7 ± 3.6). 310 participants (80.5%) were aware that combining caloric restriction with regular exercise increases energy expenditure, leading to stable weight loss. This high level of awareness likely reflects broad messaging on diet and physical activity shared by healthcare providers, media outlets, and health educational campaigns [16,17]. Additionally, 45.5% believe that gastrectomy and gastric bypass surgery are feasible options if lifestyle modifications and medications fail to manage weight. In contrast, research in China found that only 28.63% of diabetic patients were aware that surgical interventions can aid weight reduction [8]. This difference might be explained by the moderate awareness among Saudis regarding the benefits of bariatric surgeries [18]. Furthermore, only 39.0% of the patients knew the BMI ranges for overweight and obesity. These results highlight the need for public health efforts to increase awareness of BMI thresholds within the population.

The mean attitude score was 28.5 ± 4.4. A total of 78.7% individuals agreed or strongly agreed that they should put more effort into losing weight compared to the non-diabetic population. This reflects a greater understanding of the complexity of weight management, aligning with clinical guidance that stresses its multifactorial nature [19]. Additionally, only 13.8% disagreed or strongly disagreed that weight loss through dietary changes and physical activity is distressing. These findings indicate that most individuals with diabetes encounter challenges with lifestyle modifications for weight reduction.

The average practice score was 28.3 ± 4.0. A total of 24.7% of participants would always consider bariatric surgeries for weight loss if dietary interventions, regular exercise, and medications do not help, while 26.2% would frequently consider them. This can be explained by the moderate awareness in Saudi Arabia of the advantages of bariatric surgery for weight reduction and regulation [18].

Correlation analyses revealed no link between knowledge and attitudes (*r* = −0.016, *p* = 0.750). This likely occurs because the knowledge test measures facts, while the attitude scale captures feelings and perceived barriers. A weak statistically significant positive relationship between knowledge and practice was found (*r* = 0.155, *p* = 0.002). Knowledge enables basic actions, but practice also requires motivation, self-efficacy, time, cost, and support. In addition, a moderate negative correlation was detected between attitudes and practice (*r* = −0.353, *p* = 0.001). This indicates that participants who perceive more barriers toward diabetes management tend to engage less in recommended self-care practices. These results indicate that providing information alone does not effectively change behavior. Therefore, programs should combine education with behavior change methods, such as goal setting, self-monitoring, social support, and structured follow-up. Due to the cross-sectional design, interpretations should be considered preliminary.

Although this research makes a meaningful contribution to the KAP of diabetic patients regarding weight management, it has several limitations. First, it is vulnerable to recall bias because it is a cross-sectional study, as many questions depend on participants recalling their past behaviors or knowledge. Additionally, T2DM was self-reported, which introduces a risk of misclassification bias. We did not confirm the diagnosis against clinical records or contemporaneous laboratory tests. This study lacks clinical measurements such as BMI, HbA1c, and FBG levels that could be used to verify self-reported practices. Given these limitations, it is advisable to adopt longitudinal study designs to better assess changes in KAP over time and minimize recall bias. Furthermore, future research should incorporate clinical measurements to validate self-reported data and enhance the accuracy of the findings.

## 5. Conclusions

Patients with T2DM in Saudi Arabia showed moderate knowledge, negative attitudes, and moderate weight-management practices. The gap between knowing and doing highlights the need to combine education with behavior-change supports such as goal setting, self-monitoring, social support, and structured follow-up. Targeted interventions should focus on groups with lower scores and address attitudinal barriers that hinder action. Weight management programs can serve as effective entry points for broader health promotion, offering practical skills while reinforcing diabetes self-care. Integrating these components into routine clinical counseling and community initiatives may lead to more sustained, population-wide improvements.

## Figures and Tables

**Table 1:** Factors associated with diabetic patients’ knowledge, attitudes, and practices regarding weight management.

Factor		Knowledge (Mean ± SD)	*p*-Value	Attitude (Mean ± SD)	*p*-Value	Practice (Mean ± SD)	*p*-Value
Age in years	<35	7.76 ± 3.95	0.001 *	28.36 ± 4.21	0.494	28.40 ± 4.02	0.575
	35–55	9.55 ± 3.43		28.47 ± 4.48		28.33 ± 3.91	
	>55	9.35 ± 3.08		29.10 ± 5.20		27.82 ± 4.22	
Gender	Male	8.92 ± 3.68	0.314 ^	27.77 ± 4.28	0.006 *^	28.14 ± 4.34	0.627 ^
	Female	8.53 ± 3.74		29.05 ± 4.58		28.35 ± 3.79	
Educational level	Primary school or below	7.57 ± 3.92	0.163	29.33 ± 5.00	0.687	26.00 ± 4.34	0.029 *
	Junior high school/Senior high school/Technical secondary school	8.36 ± 3.61		28.58 ± 4.74		28.41 ± 3.87	
	Junior college or above	8.91 ± 3.73		28.46 ± 4.37		28.39 ± 4.01	
Medical-related occupation	Yes	9.22 ± 3.68	0.211 ^	28.36 ± 3.93	0.729 ^	28.52 ± 4.26	0.585 ^
	No	8.58 ± 3.71		28.57 ± 4.61		28.21 ± 3.97	
Physical labor occupation	Yes	8.67 ± 3.85	0.933 ^	27.91 ± 4.40	0.025 *^	28.51 ± 3.76	0.323 ^
	No	8.70 ± 3.63		28.96 ± 4.53		28.10 ± 4.18	
Monthly income in Saudi Riyal	<5000 SAR	8.16 ± 3.77	0.021 *	28.59 ± 4.48	0.667	28.29 ± 3.91	0.404
	5000–10,000 SAR	8.74 ± 3.78		28.79 ± 4.04		27.79 ± 4.01	
	10,000–20,000 SAR	9.17 ± 3.50		28.48 ± 4.75		28.42 ± 4.25	
	>20,000 SAR	10.13 ± 3.34		27.66 ± 5.27		29.12 ± 3.97	
Medical insurance	Statutory health insurance	8.34 ± 3.86	0.332	28.26 ± 4.28	0.002 *	28.48 ± 3.86	0.696
	Statutory health insurance combined with private	8.96 ± 3.49		27.22 ± 4.27		28.17 ± 4.05	
	Uninsured	8.89 ± 3.67		29.37 ± 4.65		28.11 ± 4.15	
Course of diagnosis with type 2 diabetes mellitus	Less than one year	8.36 ± 3.83	0.032 *^	28.30 ± 4.32	0.199 ^	28.57 ± 4.09	0.070 ^
	More than one year	9.18 ± 3.49		28.90 ± 4.74		27.81 ± 3.87	
Medication for blood glucose control	Oral hypoglycemic drugs	9.94 ± 2.94	0.001 *	28.41 ± 4.76	0.306	28.49 ± 4.19	0.049 *
	Injecting insulin	8.06 ± 3.59		28.93 ± 4.03		28.51 ± 3.66	
	Combined control	9.36 ± 3.74		29.64 ± 5.07		26.33 ± 4.32	
	Others	9.57 ± 2.70		29.14 ± 4.95		27.71 ± 1.50	
	None of the above	6.82 ± 4.19		27.93 ± 4.17		28.43 ± 3.91	
Diagnosed with hyperlipidemia	Yes	9.84 ± 3.11	0.018 *^	27.64 ± 4.02	0.131 ^	28.18 ± 4.32	0.873 ^
	No	8.52 ± 3.77		28.67 ± 4.56		28.28 ± 3.97	
Diagnosed with fatty liver	Yes	9.30 ± 3.43	0.152 ^	27.52 ± 3.98	0.049 *^	29.21 ± 3.44	0.042 *^
	No	8.57 ± 3.76		28.74 ± 4.57		28.08 ± 4.10	
Excess visceral fat screening	Yes	9.74 ± 3.54	0.004 *^	27.29 ± 4.11	0.005 *^	28.96 ± 3.74	0.081 ^
	No	8.41 ± 3.71		28.87 ± 4.55		28.08 ± 4.07	

P: One-way ANOVA; ^: Independent t-test; * *p* < 0.05 (significant).

**Table 2:** Pearson correlation analysis of knowledge, attitude, and practice dimensions among participants.

	Knowledge	Attitude	Practice
knowledge	1		
Attitude	−0.016 (*p* = 0.750)	1	
Practice	0.155 (*p* = 0.002 *)	−0.353 (*p* = 0.001 *)	1

*r* = Pearson correlation coefficient (two-tailed; −1 to +1), Strength (Cohen): |*r*|≈0.10 small, |*r*|≈0.30 moderate, |*r*|≈0.50 large. * Significance: *p* < 0.05.

## Data Availability

The original contributions presented in the study are included in the article and its Supplementary Material. Further inquiries can be directed to the corresponding author.

## Supplementary Materials

The following supporting information can be downloaded at https://www.mdpi.com/article/10.3390/healthcare13212770/s1, Table S1: Socio-demographic data of the study; Table S2: Diabetic patients’ knowledge regarding the role of weight management in preventing and managing diabetes; Table S3: Diabetic patients’ attitude of the role of weight management in preventing and managing diabetes; Table S4: The practices of diabetic patients regarding weight management.

## Abbreviations

The following abbreviations are used in this manuscript:

ANOVA	Analysis of Variance
BMI	Body Mass Index
DM	Diabetes Mellitus
FBG	Fasting Blood Glucose
HbA1c	Hemoglobin A1c
IRB	Institutional Review Board
KAP	Knowledge, Attitude, and Practice
N	Sample size
*P*	*p*-value (probability value)
*R*	Pearson correlation coefficient
SD	Standard Deviation
SPSS	Statistical Package for the Social Sciences
SAR	Saudi Riyal
T2DM	Type 2 Diabetes Mellitus
