# Supplementary material for: Knowledge, Attitudes, and Practices Toward Weight Management Among Patients with Type 2 Diabetes Mellitus in Saudi Arabia: A Cross-Sectional Study
Source: Healthcare (Basel). 2025 Oct 31;13(21):2770. doi: 10.3390/healthcare13212770 (PMC12607541; doi:10.3390/healthcare13212770)
Supplement: Supplementary file 1 [file healthcare-13-02770-s001.zip › healthcare-3904534-supplementary.pdf]

Table S1. Socio-demographic data of the study

Items	No	%
**Age in years**		
18-25	131	34.0%
26-35	46	11.9%
36-45	67	17.4%
46-55	69	17.9%
56-60	28	7.3%
61-70	26	6.8%
> 70	18	4.7%
**Gender**		
Male	154	40.0%
Female	231	60.0%
**Educational level**		
Primary school or below	21	5.5%
Junior high school/ Senior high school/ Technical secondary school	103	26.8%
Junior college or above	261	67.8%
**Medical-related occupation**		
Yes	64	16.6%
No	321	83.4%
**Physical labor occupation**		
Yes	154	40.0%
No	231	60.0%
**Monthly income in Saudi Riyal**		
< 5000	173	44.9%
5000-10000	96	24.9%
10000-20000	84	21.8%
> 20000	32	8.3%
**Medical insurance**		
Statutory health insurance	149	38.7%
Statutory health insurance combined with private	72	18.7%
Uninsured	164	42.6%
**Course of diagnosis with type 2 diabetes mellitus**		
Less than one year	230	59.7%
More than one year	155	40.3%
**Medication for blood glucose control**		
Oral hypoglycemic drugs	160	41.6%
Injecting insulin	88	22.9%
Combined control	36	9.4%
Others	7	1.8%
None of the above	94	24.4%
**Diagnosed with hyperlipidemia**		
Yes	50	13.0%
No	335	87.0%
**Diagnosed with fatty liver**		
Yes	63	16.4%
No	322	83.6%
**Excess visceral fat screening**		
Yes	80	20.8%
No	305	79.2%

Table S2. Diabetic patients' knowledge regarding the role of weight management in preventing and managing diabetes

**Knowledge items**	**Yes**		**No**		**Not certain**	
	**No**	**%**	**No**	**%**	**Not certain**	**%**
BMI falls within 18.5 ~ 24 kg/m2, while those above 24 are classified as overweight, and those above 28 fall into the obese category.	150	39.0%	70	18.2%	165	42.9%
Type 2 diabetes mellitus patients often experience comorbid overweight or obesity	211	54.8%	71	18.4%	103	26.8%
Weight management forms an integral part of the comprehensive care program for individuals with type 2 diabetes mellitus	260	67.5%	36	9.4%	89	23.1%
Overweight/obese patients with type 2 diabetes are at increased risk of cardiovascular disease or death, including sudden death, myocardial infarction, and stroke.	253	65.7%	40	10.4%	92	23.9%
Reducing body weight and fat content improves insulin resistance symptoms, thereby delaying the progression of pancreatic islet	294	76.4%	27	7.0%	64	16.6%
Weight reduction minimizes visceral fat deposition and enhances insulin secretion	273	70.9%	39	10.1%	73	19.0%
Restricting calories and exercising regularly boosts energy expenditure, leading to sustainable and stable weight loss results.	310	80.5%	20	5.2%	55	14.3%
Weight loss contributes to lower blood sugar, blood pressure, and lipid levels, improving overall diabetes outcomes.	308	80.0%	23	6.0%	54	14.0%
Weight control aids in achieving "diabetes mellitus in remission" status for patients with type 2 diabetes mellitus	255	66.2%	24	6.2%	106	27.5%
After reaching the overall goal or “post-diabetes remission,” maintaining weight control and undergoing regular follow-up remains essential.	287	74.5%	25	6.5%	73	19.0%
In addition to lifestyle improvements, diet pills such as orlistat and blood sugar medications such as liraglutide may help control weight in people with type 2 diabetes.	197	51.2%	28	7.3%	160	41.6%
For patients who have difficulty controlling weight through lifestyle changes and medication, gastrectomy and gastric bypass surgery are viable options.	175	45.5%	59	15.3%	151	39.2%
Weight management may no longer be necessary after attaining the combined target or "diabetes mellitus in remission	119	30.9%	155	40.3%	111	28.8%
Has your doctor discussed the weight management requirements with you	228	59.2%	103	26.8%	54	14.0%
**Overall score range**			0-13			
**Overall score Mean ± SD**			8.7 ± 3.6			

Table S3. Diabetic patients' attitude of the role of weight management in preventing and managing diabetes

Attitude	Strongly disagree		Disagree		Neutral		Agree		Strongly agree	
	**No**	**%**	**No**	**%**	**No**	**%**	**No**	**%**	**No**	**%**
Diabetes significantly increases my susceptibility to obesity compared to the general population	9	2.3%	24	6.2%	79	20.5%	115	29.9%	158	41.0%
Achieving weight control requires greater effort on my part compared to healthy individuals	7	1.8%	13	3.4%	62	16.1%	119	30.9%	184	47.8%
Medication provides a more manageable approach to weight control than exercise	7	1.8%	20	5.2%	118	30.6%	107	27.8%	133	34.5%
I prefer hypoglycemic drugs that facilitate weight loss	16	4.2%	25	6.5%	118	30.6%	103	26.8%	123	31.9%
Weight loss adversely affects my resistance levels	22	5.7%	45	11.7%	131	34.0%	100	26.0%	87	22.6%
Dieting and exercising for weight control have been distressing	18	4.7%	35	9.1%	89	23.1%	121	31.4%	122	31.7%
There are definite medication-induced side effects associated with weight loss	5	1.3%	13	3.4%	96	24.9%	144	37.4%	127	33.0%
Weight loss through surgical procedures (e.g., gastrectomy) may evoke feelings of shame	47	12.2%	46	11.9%	118	30.6%	78	20.3%	96	24.9%
I experience anxiety when people around me comment on my weight loss	35	9.1%	45	11.7%	107	27.8%	100	26.0%	98	25.5%
I perceive no need for weight loss if I only have a slight excess of visceral fat and a normal BMI	25	6.5%	44	11.4%	109	28.3%	120	31.2%	87	22.6%
**Overall score range**					19-43					
**Overall score Mean ± SD**					28.5 ± 4.4					

Table S4. The practices of diabetic patients regarding weight management

Practice	Never		Sometimes		Occasionally		Frequently		Always	
	No	%	No	%	No	%	No	%	No	%
Carbohydrate intake in my daily diet will be monitored	25	6.5%	91	23.6%	22	5.7%	120	31.2%	127	33.0%
I control my weight using methods like intermittent fasting or ketogenic diets in my daily life	30	7.8%	85	22.1%	30	7.8%	120	31.2%	120	31.2%
I occasionally have additional meals to prevent hypoglycemia	30	7.8%	95	24.7%	18	4.7%	128	33.2%	114	29.6%
I do not resist eating more when urged by family or friends	20	5.2%	109	28.3%	27	7.0%	128	33.2%	101	26.2%
Daily weight control involves engaging in resistance exercises and aero	26	6.8%	87	22.6%	23	6.0%	129	33.5%	120	31.2%
I substitute exercise with household chores and other activities	14	3.6%	89	23.1%	30	7.8%	137	35.6%	115	29.9%
I use medication (e.g., orlistat, metformin) for weight control	72	18.7%	108	28.1%	18	4.7%	97	25.2%	90	23.4%
Following medication intake, I may relax the diet and exercise requirements	32	8.3%	105	27.3%	25	6.5%	125	32.5%	98	25.5%
If lifestyle changes and medications prove ineffective in weight control, I may consider surgery	65	16.9%	93	24.2%	31	8.1%	101	26.2%	95	24.7%
**Overall score range**	15-38									
**Overall score Mean ± SD**	28.3 ± 4.0									
